# Association of *Bach1* Gene Polymorphisms with Susceptibility to Bronchopulmonary Dysplasia in Preterm Infants

**DOI:** 10.3390/biomedicines14010017

**Published:** 2025-12-21

**Authors:** Satomi Sakuraba, Atsuko Noguchi, Hirokazu Arai, Ayumi Sasaki, Mitsuhiro Haga, Ayaka Iwatani, Eri Nishimura, Nobuhiko Nagano, Shutaro Suga, Shunsuke Araki, Asami Konishi, Yoshihiro Onouchi, Masato Ito, Fumihiko Namba

**Affiliations:** 1 Department of Pediatrics, Akita University Graduate School of Medicine, Akita 010-8543, Japan; goofumo@gmail.com (S.S.); atsuko@doc.med.akita-u.ac.jp (A.N.);; 2 Department of Pediatrics, Saitama Medical Center, Saitama Medical University, Kawagoe 350-8550, Japannambaf@saitama-med.ac.jp (F.N.); 3 Department of Pediatrics and Child Health, Nihon University School of Medicine, Itabashi 173-8610, Japan; 4 Department of Pediatrics, University of Occupational and Environmental Health, Kitakyushu 807-8556, Japan; 5 Department of Public Health, Chiba University Graduate School of Medicine, Chiba 260-8670, Japan; onouchy@chiba-u.jp; 6 Department of Neonatology, Akita Red Cross Hospital, Akita 010-1495, Japan

**Keywords:** BTB and CNC homology 1, bronchopulmonary dysplasia, home oxygen therapy, premature, single-nucleotide polymorphism

## Abstract

**Background:** BTB and CNC homology 1 (*Bach1*) are transcriptional regulators involved in the oxidative response and inflammation. Although its biological functions are well characterized, the clinical impact of *Bach1* gene polymorphisms (rs2300301, rs1153285, and rs2070401) on respiratory outcomes in preterm infants remains unclear. **Methods:** This multicenter study included 212 Japanese preterm infants born at <32 weeks of gestation with birth weights <1250 g. Three *Bach1* single-nucleotide polymorphisms (SNPs; rs2300301, rs1153285, and rs2070401) were genotyped using TaqMan polymerase chain reaction (PCR). The requirements for home oxygen therapy (HOT) were compared across genotypes. Logistic regression analyses were performed after adjusting for the gestational age, sex, birth weight, and histological chorioamnionitis status. **Results:** Infants requiring HOT had a significantly lower gestational age (26 ± 1.7 weeks vs. 27 ± 2.2 weeks, *p* = 0.015) and lower birth weight (774 ± 235 g vs. 818 ± 233 g, *p* = 0.043) than those who did not. Histological chorioamnionitis was more prevalent in the HOT group (*p* = 0.022). rs2300301 was associated with HOT in univariate analysis (OR = 1.78, 95% CI: 1.20–2.04, *p* = 0.015). However, this association did not remain statistically significant after adjustment for gestational age, sex, birth weight, and histological chorioamnionitis (OR = 2.48, 95% CI: 0.90–6.80, *p* = 0.079). The rs1153285 and rs2070401 SNPs were not significantly associated with HOT expression. **Conclusions:** Our findings suggest a potential association between the *Bach1* rs2300301 polymorphism and prolonged oxygen requirement in preterm infants. Although the adjusted analysis did not confirm the statistical significance, this SNP may serve as a candidate genetic marker for respiratory morbidity. Further studies are required to validate these findings.

## 1. Introduction

Bronchopulmonary dysplasia (BPD) is one of the most common and serious complications in preterm infants, particularly in those born before 32 weeks of gestation [1]. Globally, BPD affects approximately 40–45% of infants born at <28 weeks and 20–30% of those born at <32 weeks, representing a major contributor to neonatal morbidity and long-term respiratory impairment [2]. Nationwide data from the Neonatal Research Network of Japan have demonstrated a substantial burden of BPD. According to recent multicenter cohort reports, moderate-to-severe BPD develops in approximately 30–40% of very low birth weight infants and in more than 50% of those born before 28 weeks of gestation [3]. Home oxygen therapy (HOT) refers to the continued use of supplemental oxygen after discharge in preterm infants who remain oxygen dependent despite being otherwise clinically stable. In contemporary neonatal practice, the need for HOT at discharge is widely used as a pragmatic surrogate marker for moderate-to-severe BPD because it reflects persistent respiratory insufficiency and impaired pulmonary development beyond the neonatal period [4,5]. Given this clinical relevance, BPD continues to represent a major cause of both short-term morbidity during the neonatal period and long-term respiratory sequelae throughout childhood and beyond, emphasizing the substantial clinical and societal impact of this chronic lung disorder as the survival rates of extremely preterm infants continue to improve worldwide. Despite remarkable advances in neonatal intensive care, including improvements in mechanical ventilation strategies, surfactant replacement therapy, and antenatal steroid administration, BPD continues to be associated with substantial long-term respiratory morbidity, frequent hospital readmissions, impaired lung function, and an increased risk of neurodevelopmental impairment extending into later childhood and even adulthood [6,7,8]. The identification of reliable biological markers that can predict respiratory outcomes is crucial not only for optimizing individualized clinical management during the neonatal period but also for guiding long-term follow-up strategies, facilitating early interventions, and ultimately improving the overall quality of life in preterm infants at risk of developing BPD.

The pathogenesis of BPD is multifactorial and involves intrinsic factors, such as lung immaturity, impaired alveolar development, and underdeveloped antioxidant defenses, as well as extrinsic factors, including prolonged oxygen exposure, mechanical ventilation, and inflammatory or infectious insults during the neonatal period. Immature lungs have underdeveloped antioxidant defenses, rendering them particularly susceptible to oxygen-induced injury and oxidative stress, which can disrupt normal alveolar and vascular development, exacerbate inflammatory responses, and contribute to the progression of chronic lung diseases in preterm infants [9,10].

Oxidative stress has long been recognized as a pivotal driver of BPD [11]. Preterm infants are frequently exposed to supplemental oxygen and invasive ventilation, both of which markedly increase the production of reactive oxygen species (ROS). ROS can damage proteins, lipids, and nucleic acids, leading to impaired alveolarization, vascular rarefaction, and long-term alterations in the lung structure. Under physiological conditions, a coordinated network of antioxidant enzymes, including superoxide dismutase, catalase, glutathione peroxidase, and glutamate-L-cysteine ligase, neutralizes ROS and preserves redox homeostasis [12,13]. However, these enzymatic defenses are developmentally immature in preterm infants, making the lungs particularly vulnerable to oxidative injury. Insights from neonatal rodent and large-animal models of hyperoxia have further demonstrated that reduced antioxidant capacity exacerbates alveolar simplification, inflammation, and vascular remodeling, thereby establishing oxidative stress as a central mechanism in the evolution of “new BPD” [14,15].

Apart from environmental factors such as mechanical ventilation, prolonged oxygen exposure, and perinatal inflammatory insults, genetic predisposition is increasingly recognized as an important modifier of disease susceptibility and severity, influencing individual variability in clinical outcomes, response to therapy, and long-term respiratory trajectories among preterm infants with BPD [16].

Over the past few decades, numerous studies have investigated the contribution of genetic variations to the risk of BPD. Candidate gene approaches have implicated polymorphisms in oxidative stress-related enzymes, inflammatory mediators, vascular growth regulators, and surfactant proteins as potential modifiers of susceptibility [17,18,19,20,21]. Importantly, many of these associations remain in the exploratory stage, as functional validation is limited and large-scale multi-ethnic cohorts are still lacking. Collectively, these studies underscore the importance of the host genetic background in shaping neonatal outcomes, while highlighting the need to investigate additional candidate genes with strong biological plausibility.

Among the key molecular pathways implicated in oxidative lung injury is the nuclear factor erythroid 2-related factor 2 (Nrf2)–heme oxygenase-1 (HO-1) signaling axis, which serves as a central defense mechanism against ROS by regulating the transcription of multiple antioxidant and cytoprotective genes, thereby playing a pivotal role in maintaining redox balance and limiting tissue damage in the developing lung. This pathway plays a central cytoprotective role in mitigating oxidative stress by activating downstream antioxidant enzymes, maintaining cellular redox homeostasis, and reducing oxidative damage to alveolar and vascular structures, thereby contributing to the preservation of normal lung growth and function in vulnerable preterm infants [22]. Bach1, a BTB and CNC homology 1 transcription factor, functions as a key repressor of *HO-1* and other antioxidant genes by competing with Nrf2 to bind to antioxidant response elements, thereby suppressing the transcriptional activation of cytoprotective pathways and exacerbating cellular vulnerability to oxidative stress in immature lungs [23]. In neonatal animal models, HO-1 plays a critical role in lung development [24], promotes tissue repair and regeneration following hyperoxic injury [25], and regulates vascular formation and remodeling within developing lungs [26]. In contrast, Bach1 deficiency accelerates recovery from hyperoxia-induced lung injury by enhancing antioxidant defenses and attenuating inflammatory responses [27,28], thereby highlighting the pivotal balance between Bach1 and HO-1 signaling in pulmonary homeostasis.

Studies have begun to explore the role of genetic polymorphisms in the expression of oxidative stress–related genes on neonatal outcomes [18,29,30,31]. However, the potential contribution of the *Bach1* gene remains largely unexplored, particularly in the context of preterm infant populations where oxidative stress plays a central role in disease pathogenesis.

In this multicenter study, we investigated whether three single-nucleotide polymorphisms (SNPs) in *Bach1* (rs2300301, rs1153285, and rs2070401)—including one missense variant (rs2300301) and two intronic variants (rs1153285 and rs2070401)—were associated with the need for home oxygen therapy (HOT) at the point of discharge, a widely used surrogate marker of moderate-to-severe BPD that reflects persistent respiratory insufficiency and impaired pulmonary development.

## 2. Materials and Methods

### 2.1. Study Design and Patient Groups

**Figure 1:**
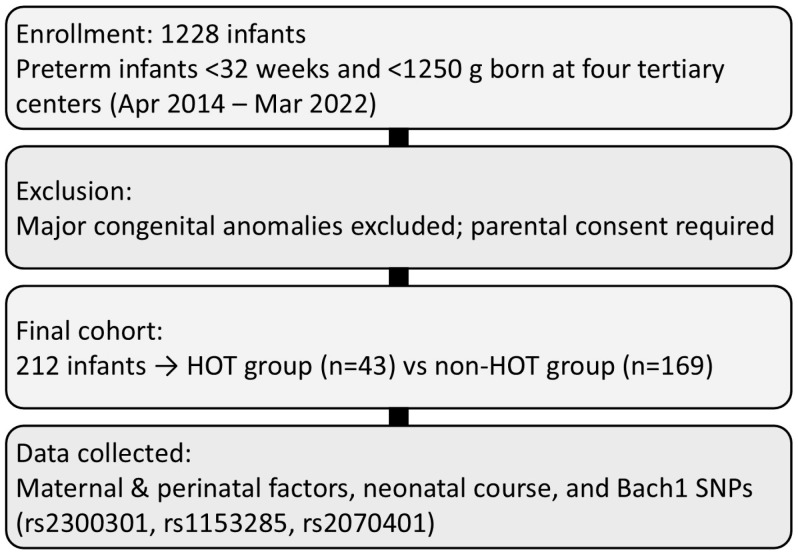
Flow diagram of patient enrollment and selection for genetic analysis of Bach1 polymorphisms.

In this study, we included 212 preterm infants born at a gestational age (GA) < 32 weeks and birth weight (BW) < 1250 g. During the study period, a total of 1228 potentially eligible infants born at a GA of <32 weeks with birth weights < 1250 g were admitted to the four participating tertiary centers—Saitama Medical Center (SMC; n = 519), Nihon University Itabashi Hospital (NUIH; n = 315), University of Occupational and Environmental Health (UOEH; n = 234), and Akita Red Cross Hospital (ARCH; n = 160). Infants with major congenital anomalies were excluded before the informed consent process. Genetic analysis was then performed only for infants whose parents provided consent, resulting in a final cohort of 212 infants included in the present study. The infants were subsequently classified into two groups according to their respiratory status at discharge: newborns who required home oxygen therapy (HOT group, n = 43) and those who were discharged without the need for supplemental oxygen (non-HOT group, n = 169). Clinical characteristics, including perinatal factors and neonatal course, together with Bach1 genetic polymorphism data, were systematically compared between the HOT and non-HOT groups to evaluate potential associations between genotype distribution and the severity of respiratory outcomes (Figure 1).

### 2.2. Patient Clinical Data

The clinical data analyzed in this study were obtained from the medical records of the patients and included detailed information on maternal background, perinatal factors, and neonatal clinical course. The data included maternal age, antenatal steroid therapy, histological chorioamnionitis, mode of delivery, GA, BW, sex, Apgar scores at 1 and 5 min, use of surfactants, diagnosis of BPD according to the 2016 National Institute of Child Health and Human Development definition [32], and HOT at discharge. Across the participating centers, HOT initiation did not follow a single standardized criterion. Instead, a common Japanese clinical practice was applied: infants who remained oxygen-dependent but were otherwise medically stable and eligible for discharge after approximately 40 weeks of corrected age were discharged with HOT. Although some centers used SpO_2_ thresholds, such as difficulty maintaining a 24 h average saturation of approximately 95%, minor variations existed in monitoring practices and clinical judgment. In this study, infants discharged with supplemental oxygen were uniformly categorized into the HOT group, enabling consistent analysis despite these subtle inter-institutional differences and reflecting real-world clinical decision-making across Japanese neonatal units. The initiatives for data collection commenced sequentially at each participating facility on 4 January 2016, and concluded between 2 April and 24 April 2022. Data collection specifically concluded on 24 April 2022 at SMC, 2 April 2022 at NUIH, 5 April 2022 at UOEH, and 14 April 2022 at ARCH, thereby ensuring a consistent multicenter dataset that spanned more than six years of clinical practice.

Genomic DNA was extracted from peripheral blood samples obtained from the infants using the Wizard^®^ Genomic DNA Purification Kit (Promega, Madison, WI, USA), according to the manufacturer’s protocol, to ensure that the DNA was suitable for subsequent genotyping analyses. Three single nucleotide polymorphisms (SNPs) (s2300301, rs1153285, and rs2070401) surrounding *Bach1* (NM_001186) were selected. These SNPs have previously been reported to be associated with oxidative stress–related conditions, including susceptibility to anti-TB drug-induced hepatotoxicity in Asian populations [33], suggesting their potential relevance beyond pulmonary disease. The genomic location of the *Bach1* gene is described with reference to the GRCh38.p14 genome assembly, which is located on chromosome 21 (NC_000021.9), specifically in the 21q22.11 region. rs2300301 and rs1153285 are positioned in intron 1, whereas rs2070401 is in the 3′-UTR. The selected SNPs generally exhibited minor allele frequencies of approximately 0.10 in the Japanese population, with rs1153285 and rs2070401 meeting the threshold, and rs2300301 slightly below the threshold. For genotyping, real-time polymerase chain reaction (PCR) was performed using an ABI 7900HT PCR device together with TaqMan SNP assays (Applied Biosystems, Foster City, CA, USA), following standard protocols to ensure accurate and reproducible detection of targeted polymorphisms.

### 2.3. Statistical Analysis

This was a retrospective secondary analysis of an existing multicenter cohort; therefore, an a priori sample size calculation was not performed. The sample size was determined based on the number of eligible infants enrolled during the study period. Given the exploratory nature of the study, all available cases were included to maximize statistical power and precision and to ensure that potential genetic associations, particularly those involving less frequent alleles, could be assessed as comprehensively as possible within the constraints of the cohort.

The participants were categorized into genotype groups according to the number of minor alleles present at each SNP location, allowing the assessment of potential dose-dependent genetic effects. Descriptive statistics are summarized as medians with interquartile ranges for continuous variables and as frequencies and percentages for categorical variables to provide a comprehensive overview of the baseline characteristics. Comparisons between the HOT and non-HOT groups were conducted using the chi-square test or Fisher’s exact test for categorical variables and the Mann–Whitney U test or Kruskal–Wallis test for continuous variables, as appropriate, thereby ensuring that the statistical methods matched the distributional properties of the data.

To evaluate the association between *Bach1* genetic variants and risk of HOT at discharge, both univariate and multivariate logistic regression analyses were performed. The multivariate model incorporated clinical variables that demonstrated significant group differences in univariate comparisons, specifically gestational age, sex, and histological chorioamnionitis, to adjust for potential confounding factors. The results of the regression analyses were presented as odds ratios (ORs) with corresponding 95% confidence intervals (CIs), providing estimates of both the strength and precision of the associations.

All statistical analyses were conducted using a two-tailed approach, with a *p*-value of <0.05 considered statistically significant. Statistical computations were performed using SPSS software (version 29.0; IBM, Armonk, NY, USA) to ensure standardized and reproducible procedures. Linkage disequilibrium (r^2^) between *Bach1* SNPs was additionally assessed using Haploview (version 4.2; Broad Institute, Cambridge, MA, USA) [34] to explore potential non-random associations among the polymorphisms.

## 3. Results

A total of 212 preterm infants who met the eligibility criteria were enrolled from the four participating medical institutions (Table 1). The HOT group (n = 43) had a significantly lower gestational age (26 ± 1.7 weeks) and birth weight (774 ± 235 g) compared to the non-HOT group (27 ± 2.2 weeks, *p* = 0.015; 818 ± 235 g, *p* = 0.043), indicating that infants who ultimately required HOT were generally more premature and smaller at birth than those discharged without supplemental oxygen. There were no significant differences between the two groups with respect to maternal age, Apgar scores at 1 and 5 min, sex distribution, antenatal steroid administration, or use of surfactant therapy, suggesting comparable baseline characteristics across these variables. However, the incidence of histological chorioamnionitis was significantly higher in the HOT group than in the non-HOT group (58% vs. 36%, *p* = 0.022), indicating a possible contributory role of perinatal inflammation in the increased risk of HOT (Table 2).

**Figure 2:**
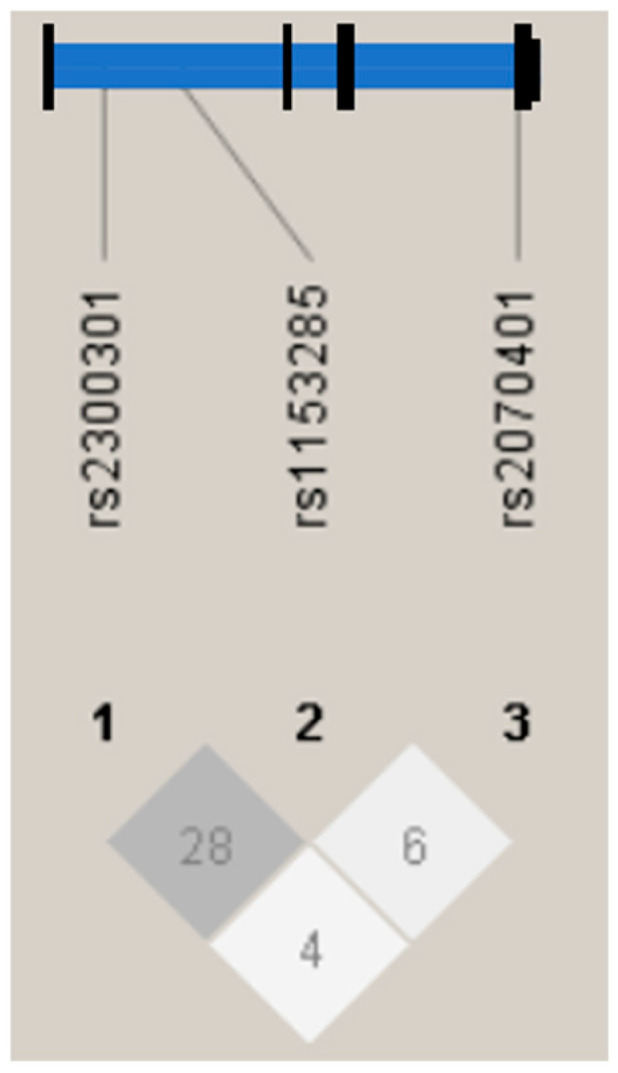
Genomic organization and linkage disequilibrium mapping of single-nucleotide polymorphisms (SNPs) in *Bach1*. The upper diagram shows the structure of *Bach1* and relative physical position of three SNPs. The pairwise linkage disequilibrium (r2) between the SNPs is shown below each SNP combination.

The genotype distributions for rs2300301, rs1153285, and rs2070401 conformed to the Hardy–Weinberg equilibrium, confirming the reliability of the genotyping data. Linkage disequilibrium analysis showed a low-to-moderate correlation between rs2300301 and rs1153285 (r^2^ = 0.28), whereas rs2070401 appeared to be largely independent of the other two loci (r^2^ = 0.06 and 0.04) (Figure 2), suggesting a minimal allelic association. Consistent with these findings, no strong haplotype block structures were observed among the three SNPs, indicating that they were segregated independently within this cohort.

Among the analyzed SNPs, rs2300301 was significantly associated with the requirement for HOT at discharge in univariate analysis (Table 3). The genotype distribution at this locus was as follows: GG in 76 infants, GA in 86 infants, and AA in 50 infants. Infants carrying the GG or GA genotypes demonstrated a significantly higher frequency of HOT use compared to those with the AA genotype (OR = 1.78, 95% CI: 1.20–2.04, *p* = 0.015), suggesting that the presence of the G allele may contribute to increased vulnerability to respiratory insufficiency. In contrast, no significant association was observed between HOT and rs1153285 or rs2070401, indicating that their influence on BPD severity was limited in this cohort.

To further clarify the allele-level effect suggested by the genotype distribution, we calculated the allele counts for the three Bach1 SNPs and examined their associations with HOT requirements. The results are summarized in Table 4. Consistent with the genotype-based findings, the G allele of rs2300301 was significantly more frequent in infants requiring HOT than in those not requiring HOT (*p* = 0.048), supporting a potential dose-dependent genetic influence. In contrast, no significant allele-level associations were observed for rs1153285 or rs2070401, suggesting that the functional relevance of this genomic region is primarily driven by rs2300301 or its variants.

Subgroup analyses were conducted to determine whether key clinical variables modified the association between rs2300301 and HOT (Table 5). Even after stratification by factors, such as antenatal steroid use, surfactant administration, and mode of delivery, the association between rs2300301 and HOT remained directionally consistent across subgroups, suggesting a potential independent genetic contribution that was not substantially confounded by these perinatal interventions.

In multivariate logistic regression analysis adjusting for gestational age, sex, and histological chorioamnionitis (Table 6), the GG/GA genotype of rs2300301 demonstrated a trend toward an increased risk of requiring HOT at discharge (OR = 2.48, 95% CI: 0.90–6.80, *p* = 0.079), although this association did not reach the threshold for statistical significance. Similarly, none of the evaluated clinical or genetic factors, including rs2300301 genotype, gestational age, histological chorioamnionitis, or male sex, showed statistically significant associations in the multivariate model, underscoring the multifactorial nature of BPD.

## 4. Discussion

Among the three *Bach1* SNPs analyzed, rs2300301 demonstrated a significant association with HOT requirement in univariate analysis. Infants carrying the GG or GA genotype were more likely to require supplemental oxygen therapy at discharge than those carrying the AA genotype, highlighting the possible role of the G allele in conferring increased vulnerability. Although this association was not statistically significant after adjusting for gestational age, sex, and histological chorioamnionitis in the multivariate model, point estimates suggested a possible but inconclusive association. Taken together, these findings suggest that *Bach1* genetic variation, particularly rs2300301, may contribute to susceptibility to prolonged respiratory support in preterm infants, potentially through the modulation of antioxidant defenses and inflammatory pathways, and may serve as a candidate marker for identifying high-risk populations in future precision medicine approaches.

The biological plausibility of this association can be explained by the genomic context of rs2300301. In our study, rs2300301 showed a trend toward an increased risk of severe BPD and/or the requirement for home oxygen therapy, although this association did not remain statistically significant after adjustment for clinical covariates. Rs2300301 is located in intron 1 of Bach1, a region that may harbor regulatory elements controlling gene expression. Intronic variants can influence pre-mRNA splicing, transcription factor binding, or chromatin architecture; therefore, rs2300301 or variants in linkage disequilibrium with it may modulate Bach1 expression and downstream antioxidant pathways, including the Nrf2–HO-1 axis. Such alterations in oxidative stress regulation and inflammatory signaling could partly explain the observed trend toward worse respiratory outcomes among infants carrying the A allele. In addition, rs2300301 shows modest linkage disequilibrium with neighboring Bach1 variants, raising the possibility that it serves as a proxy marker for other functional regulatory polymorphisms within this locus.

The minor allele frequency of rs2300301 in our cohort (A allele: 0.439) closely matched that in the East Asian population reported for gnomAD (A allele: 0.44). This concordance indicates that our sample is representative of the broader Japanese population, and supports the validity of the observed genetic association. The similarity in allele distribution also suggests that the association is unlikely to be due to sampling distortion but rather reflects a potentially meaningful link between impaired antioxidant regulation and prolonged oxygen dependency in preterm infants. Taken together, these characteristics provide further justification for continued investigation of Bach1-related pathways in neonatal lung disease.

In addition, preterm infants who required HOT had a lower gestational age and birth weight, and a higher incidence of histological chorioamnionitis than those who did not require supplemental oxygen at discharge. Although these clinical differences are consistent with well-established risk factors for BPD development, they appear to be less predictive of prolonged oxygen dependency than the genetic trends observed for rs2300301. This suggests that although traditional perinatal and clinical factors continue to play an important role in shaping respiratory outcomes, *Bach1* genetic variation may provide additional explanatory power beyond these factors and could complement existing risk stratification approaches in preterm populations.

Ferroptosis, an iron-dependent form of regulated cell death driven by lipid peroxidation, has recently been recognized as a key mechanism that contributes to hyperoxia-induced lung injury and impaired alveolar development in preterm infants. Bach1 plays a central role in this process by repressing antioxidant and cytoprotective genes, including HO-1, GCLC, and FTH1, thereby limiting the activation of the Nrf2–HO-1 axis and reducing cellular resilience to oxidative injury [22,23,35,36,37]. In neonatal models, HO-1 contributes to lung maturation, tissue repair, and angiogenesis [24,25,26], while Bach1 deficiency accelerates recovery from hyperoxic injury by enhancing the antioxidant capacity and reducing inflammation [27,28]. Bach1 also promotes ferroptosis by suppressing glutathione synthesis and limiting GPX4 activity [37], increasing vulnerability to lipid peroxidation in a highly oxidative premature lung environment. Furthermore, Bach1 has been implicated in profibrotic and proinflammatory signaling via extracellular signal-regulated kinase (ERK) activation, and its pharmacological or genetic inhibition ameliorates lung fibrosis and inflammatory injury in vivo [38]. Beyond the lung, *Bach1* variants have been linked to susceptibility to antituberculous drug-induced hepatotoxicity [39] and IgA nephropathy [40], highlighting their systemic relevance. Collectively, these mechanistic insights provide a strong biological rationale for examining Bach1 polymorphisms in preterm respiratory morbidity and suggest that modulation of the Bach1–HO-1 axis—either by enhancing Nrf2-driven antioxidant responses or relieving Bach1-mediated repression—may represent a potential therapeutic strategy for preventing BPD progression and long-term pulmonary impairment, particularly in infants with genetic backgrounds conferring heightened vulnerability.

Beyond the lung, *Bach1* genetic variants have also been associated with susceptibility to antituberculous drug-induced hepatotoxicity [39] and IgA nephropathy [40], highlighting their potential utility as genetic markers of disease susceptibility across different organ systems and underscoring the importance of considering *Bach1* variation in both pulmonary and systemic disease contexts. Preclinical studies have emphasized the clinical relevance of this pathway. The activation of the Nrf2–HO-1 axis has been shown to mitigate hyperoxic lung injury, preserve alveolar and vascular development, and protect against the onset of BPD in animal models [22]. These findings suggest that the modulation of the Bach1–HO-1 axis, either by enhancing Nrf2-driven antioxidant responses or by inhibiting Bach1-mediated repression, could represent a promising therapeutic strategy for preterm infants at risk of BPD, offering a biologically plausible approach to reduce oxidative damage and improve long-term pulmonary outcomes.

Our findings suggest a potential association between *Bach1* rs2300301 polymorphism and HOT requirement at discharge in preterm infants. Although this association did not reach statistical significance after adjusting for clinical covariates, the observed trend highlights the complex interplay between genetic susceptibility and established clinical risk factors in shaping respiratory outcomes. Importantly, this study provides novel insights into the role of oxidative stress–related genetic variation in neonatal respiratory morbidity, underscoring the potential contribution of Bach1 to the pathogenesis of BPD and emphasizing the need for larger, prospective investigations to validate and extend these preliminary findings.

This study has several limitations that should be considered when interpreting the results. Firstly, although the total number of potentially eligible infants during the study period was known, genetic analysis was performed only in infants whose caregivers provided informed consent. As a substantial proportion of eligible infants did not undergo genotyping owing to a lack of consent, the final study population may not fully represent the overall eligible cohort, leading to a potential selection bias. Secondly, the small number of infants requiring HOT (n = 43) limited the statistical power of the adjusted analysis, preventing definitive conclusions regarding the effect size of rs2300301 and increasing the likelihood of a type II error. Thirdly, the criteria for initiating and discontinuing HOT were not fully standardized across centers. Although all institutions discharged infants who remained oxygen-dependent but were otherwise stable after approximately 40 weeks of corrected age, specific SpO_2_ thresholds and monitoring strategies varied, which may have introduced heterogeneity into the outcome definition and influenced clinical decision-making. Additional unmeasured center-level practices may have also contributed to the variability in HOT assignments. Fourthly, the sample was restricted to the Japanese population; thus, the findings may not be generalizable to other ethnic or geographic groups where allele frequencies and environmental exposures differ. Fifthly, although we assessed three *Bach1* SNPs, other potentially relevant variants, including those in regulatory regions or linked pathways, were not evaluated. Sixthly, only clinical outcomes at discharge were analyzed, and long-term respiratory or neurodevelopmental outcomes were not available, restricting our ability to assess the broader implications of *Bach1* polymorphisms. Finally, functional validation of these variants was not performed; therefore, the biological mechanisms underlying the observed associations remain unclear. Therefore, future studies should aim to recruit larger, ethnically diverse cohorts, apply standardized definitions of HOT and BPD severity, and incorporate long-term follow-up to capture persistent respiratory morbidity. In addition, mechanistic studies, including in vitro and in vivo functional assays of *Bach1* variants, are essential to elucidate their roles in oxidative stress regulation and neonatal lung development.

In conclusion, this multicenter study of Japanese preterm infants suggests a possible association between the *Bach1* rs2300301 SNP and the requirement of HOT as a marker for severe BPD. Although the observed association was not statistically significant after adjusting for clinical variables, the consistent trend underscores the potential role of Bach1 genetic variation in shaping respiratory outcomes. These findings add to the growing body of evidence that polymorphisms in oxidative stress-related pathways may underlie inter-individual differences in disease susceptibility, influencing both vulnerability to lung injury and the ability to recover. Importantly, they highlighted the need to integrate genetic information with established clinical predictors to refine risk stratification and move toward precise neonatal care, while encouraging future studies to incorporate functional assays and longitudinal follow-up to clarify the mechanistic relevance of Bach1 variation and its impact on long-term pulmonary development. Moreover, expanding such investigations to multi-ethnic cohorts may help determine whether the observed genetic trends are universally applicable or specific to populations with distinct allele distributions.

## Figures and Tables

**Table 1:** Demographic and clinical characteristics of 212 patients.

Clinical Characteristic	SMC (n = 143)	NUIH (n = 34)	UOEH (n = 16)	ARCH (n = 19)
Gestational age, mean ± SD, weeks	26 ± 2.20	27 ± 1.8	27 ± 2.1	25 ± 1.5
Birth weight, mean ± SD, g	795 ± 232	1002 ± 1690	787 ± 265	752 ± 217
Male, n (%)	075 (52)	13 (38)	08 (50)	07 (37)
Cesarean section, n (%)	130 (91)	33 (97)	09 (56)	15 (79)
Death prior to 36 weeks PMA, n (%)	000 (0)	00 (0)	00 (0)	00 (0)
Moderate or Severe BPD, n (%)	087 (61)	30 (88)	12 (75)	16 (84)
HOT at discharge, n (%)	023 (16)	08 (24)	06 (38)	06 (32)

SMC, Saitama Medical Center; NUIH, Nihon University Itabashi Hospital. UOEH, University of Occupational and Environmental Health; ARCH, Akita Red Cross Hospital. SD, standard deviation; PMA, postmenstrual age; BPD, bronchopulmonary dysplasia. HOT: home oxygen therapy.

**Table 2:** Demographic and clinical characteristics of HOT and non-HOT patients.

Clinical Characteristic	HOT	non-HOT	*p*-Value
	(n = 43)	(n = 169)	
Maternal age, mean ± SD	33 ± 5.0	33 ± 5.2	<0.39
Antenatal steroids, n (%)	25 (58)	107 (63)	<0.59
Histological chorioamnionitis (%)	25 (58)	061 (36)	0.022
Cesarean section, n (%)	37 (86)	150 (89)	<0.54
Gestational age, mean ± SD, wk	26 ± 1.7	27 ± 2.2	0.015
Birth weight, mean ± SD, g	774 ± 235	818 ± 233	< 0.043
Male, n (%)	24 (56)	79 (47)	<0.23
Apgar score at 1 min	4(1–8)	4(0–8)	<0.27
Apgar score at 5 min	6(3–9)	7(1–10)	<0.28
Use of surfactant, n (%)	38 (88)	140 (83)	<0.49
Moderate or Severe BPD, n (%)	42 (98)	103 (61)	<0.01

SD, standard deviation; BPD, bronchopulmonary dysplasia, HOT, home oxygen therapy.

**Table 3:** The association of each genotype in and near the *Bach1* gene with home oxygen therapy at discharge.

SNPs	Genotypes	HOT (n = 43)		non-HOT (n = 169)		OR *	95% CI	*p* Value **
		n	Freq	n	Freq			
rs2300301	GG	23	0.53	53	0.31	1.78	1.20–2.04	0.015
	GA	15	0.35	71	0.42			
	AA	5	0.12	45	0.27			
rs1153285	CC	17	0.40	72	0.43	0.63	0.70–3.61	Not significant
	CT	16	0.37	70	0.41			
	TT	10	0.23	27	0.16			
rs2070401	AA	26	0.60	117	0.69	0.79	0.43–3.66	Not significant
	AG	12	0.28	36	0.21			
	GG	5	0.12	16	0.10			

OR, odds ratio; CI, confidence interval; HOT, home oxygen therapy; Freq, frequency. * Odds ratios were calculated in an assumption that underlined genotypes increase risk for HOT. ** *p* values were obtained using descriptive statistics among the three genotype groups.

**Table 4:** Allele counts of *Bach1* polymorphisms and their association with home oxygen therapy.

SNPs	Allele	HOT (n = 43)	non-HOT (n = 169)	Total	*p* Value
rs2300301	A	25	161	186	0.048
	G	61	177	238	
rs1153285	C	50	214	264	0.421
	T	36	124	160	
rs2070401	A	64	270	334	0.386
	G	22	68	90	

HOT, home oxygen therapy.

**Table 5:** Comparison of patient background and clinical characteristics of different genotypes (GG/GA genotype and AA genotype) of single nucleotide polymorphism (SNP) in intron 1 (rs2300301) of *Bach1* gene.

Clinical Characteristic	GG/GA (n = 162)	AA (n = 50)	*p*-Value
Maternal age, mean ± SD	33 ± 5.2	33 ± 5.3	0.84
Antenatal steroids, n (%)	99 (61)	33 (66)	0.53
Histological chorioamnionitis (%)	71 (44)	17 (34)	0.22
Cesarean section, n (%)	142 (88)	45 (90)	0.65
Gestational age, mean ± SD, wk	26 ± 2.2	27 ± 2.1	0.36
Birth weight, mean ± SD, g	800 ± 236	815 ± 237	0.70
Male, n (%)	79 (49)	25 (50)	0.88
Apgar score at 1 min	4(0–8)	4(0–8)	0.68
Apgar score at 5 min	7(1–10)	7(3–9)	0.85
Use of surfactant, n (%)	138 (85)	040 (80)	0.38
Moderate or Severe BPD, n (%)	112 (69)	032 (64)	0.50
Home oxygen therapy at discharge, n (%)	38 (23)	005 (10)	0.04

SD, standard deviation; BPD, bronchopulmonary dysplasia.

**Table 6:** Multivariate logistic regression analysis of HOT risk factors.

Risk Factors	OR	95% CI	*p*-Value
Lower gestational age	1.19	0.99–1.42	0.051
Male	1.68	0.83–3.40	0.146
Histological chorioamnionitis	1.25	0.93–3.83	0.080
GG or GA genotypes at rs2300301	2.48	0.90–6.80	0.079

OR, odds ratio; CI, confidence interval.

## Data Availability

The original contributions presented in this study are included in the article. Further inquiries can be directed to the corresponding authors.
